# Characterization and application of a coating of starch extracted from avocado (*Persea americana* L. cv. Hass) seeds as an alternative to reduce acrylamide content in French fries

**DOI:** 10.1007/s10068-022-01140-w

**Published:** 2022-08-12

**Authors:** O. Vega-Castro, M. Ramírez, L. Blandón-Mena, J. Contreras-Calderón, M. Mesías, C. Delgado-Andrade, F. J. Morales, D. Granda-Restrepo

**Affiliations:** 1 BIOALI Research Group, Food Department, Faculty of Pharmaceutical and Food Sciences, University of Antioquia, Street 67 No. 53-108, Medellín, Colombia; 2 Professor American University Corporation, Career. 42 No. 52-06 (Av. La Playa), Medellín, Colombia; 3 Institute of Food Science, Technology and Nutrition (ICTAN-CSIC), Madrid, Spain

**Keywords:** Avocado seed starch, French fry, Acrylamide, Drying, Coating

## Abstract

The starch extracted from avocado (*Persea americana* L. cv. Hass) seeds was characterized and used in the preparation of an edible coating to reduce the oil uptake and acrylamide content in French fries. Starch characterization was carried out using Differential Scanning Calorimetry, Fourier transform infrared spectrophotometry, gelatinization, and scanning electron microscopy. Uncoated (UFF) and coated (CFF) French fries were compared and evaluated for moisture, water activity (Aw), fat, color, firmness, acrylamide content, and sensorial analysis. The extracted starch presented a high crystalline structure and good stability to mechanical work and heat treatments. The CFF French fries showed significantly higher Aw, color parameter a*, but lower luminosity and acrylamide content than UFF samples. Similarly, the CFF samples tended to decrease the fat content, although without statistical differences. Avocado seed starch can be an economical and technically feasible alternative to the food industry as an effective coating to reduce acrylamide content in French fries.

## Introduction

Avocado (*Persea americana* Mill.) is a popularly consumed tropical and subtropical fruit considered a healthy food. It has more than 25 essential nutrients such as vitamins, minerals, proteins, fibers, and lipids. Due to its high nutritional value and health benefits, avocado cultivation and consumption have increased rapidly in recent years (Wang et al., 2019). According to FAO (2017), world avocado production was 5.9 million tons/year in 2017, with the major producing countries being Mexico, Dominican Republic, Peru, Indonesia, Colombia, and Brazil. In Colombia, the national avocado production was 544 thousand tons/year in 2018. With a planted area of approximately 56,000 hectares, 94% of the production supplies the local market, and the remaining 6% is destined for export (Minagricultura, 2019).

The growth of avocado fruit’s national and international trade generates high surpluses since not all the harvested fruit reaches export standards due to post-harvest deterioration (mechanical injury, physiological deterioration, and microbiological decay) (Bill et al., 2014). Likewise, the industrial transformation processes of the fruit generate a large amount of waste that is approximately 45% of world production. The waste is roughly distributed as 65% for the pulp, 17% for the seed, and 18% for the peel (Saavedra et al., 2017). This fact involves an economic and environmental problem for avocado producing companies (Saavedra et al., 2017). Residues from the avocado industry are an important source of carbohydrates, fiber, hemicellulose, starch, and phenolic compounds with antioxidant properties. In this sense, several authors have reported the extraction of bioactive compounds present in avocado seeds (Figueroa et al., 2018), including the technological and chemical characterization of fibers (Barbosa-Martín et al., 2016) and starch (Builders et al., 2010; Lacerda et al., 2014; Chel-Guerrero et al., 2016).

Starch is a renewable, naturally occurring carbohydrate in the form of semi-crystalline granules and is composed of amylose and amylopectin (Chen et al., 2020). One of the most referenced applications of starch is the development of edible coatings due to its ability to form a continuous matrix. An edible coating can be defined as a thin layer made up of edible materials with a primary packaging function, thus preventing physicochemical and microbiological food deterioration (Eom et al., 2018). The development of edible coatings from corn, rice, oats, cassava, yam, or potato starches has been previously reported (Singh, et al., 2020). These coatings have been used to improve the shelf life of meat products (Umaraw et al., 2020), cheeses (Berti et al., 2019), cakes and bread (Eom et al., 2018), and fruits (Maringgal et al., 2020).

Moreover, coatings have been applied in foods intended to fry, acting as a barrier in the absorption of fat or oil and reducing the formation of acrylamide (Trujillo-Agudelo et al., 2019). This last application is important from a nutritional point of view since a high consumption of fried foods is associated with a risk factor for health (WHO, 2018) and, on the other hand, acrylamide exposure has been related to the development of different types of cancer (EFSA, 2015). Acrylamide is a chemical process contaminant formed when foods containing free asparagine and reducing sugars are cooked at temperatures above 120 °C in low moisture conditions. To reduce the presence of acrylamide in foods, the European Commission has published the Regulation (EU) 2017/2158, which establishes mitigation measures and reference levels in different food groups, including French fries (European Commission, 2017).

Several investigations have described the application of coatings with different compositions to potatoes prior to the frying process to reduce the fat and/or the acrylamide content in French fries (Mousa, 2018; Trujillo-Agudelo et al., 2019). However, nowadays, coatings containing avocado by-products and their effects on the properties of the final product have not been evaluated. The objective of this work was to study the drying of avocado seeds, the starch extraction and its characterization. And, consider its application as an edible coating in potatoes to decrease acrylamide formation and oil absorption during frying. Additionally, the textural, physicochemical, and sensory properties of French fries were evaluated. The final objective was to provide an alternative strategy to promote avocado by-products, alleviate the pollution problems generated by the waste, and develop strategies in obtaining foods that favor consumer health as an alternative to global public health policies.

## Material and methods

### Samples and reagents

Avocado (*Persea Americana* L. var. Hass) seeds were supplied by Terravocado S.A. (Medellín-Colombia). The fresh potato tubers (*Solanum tuberosum* L., var. R12 Diacol Capiro) were supplied by the Instituto Colombiano Agropecuario (ICA, Colombia). The commercial fresh potatoes and palm oil (Oleollano, Villavicencio-Meta, Colombia; Lot L24082020F2TC YP) were purchased from a local market (Medellín, Colombia). The commercial French fries were purchased in a street food establishment (Medellín, Colombia). Acrylamide standard (99%), potassium hexacyanoferrate (II) trihydrate (98%, Carrez-I), and zinc acetate dehydrate (> 99%, Carrez-II) were obtained from Sigma (St. Louis, MO, USA). ^13^C_3_-labelled acrylamide (99% isotopic purity) was obtained from Cambridge Isotope Laboratories (Andover, MA, USA). Formic acid (98%), methanol (99.5%), sulfuric acid (97%), phenol, anhydrous glucose and hexane were from Panreac (Barcelona, Spain). Sodium bisulfite was from Merck (Darmstadt, Germany) and glycerol from Bell Chem International S.A.S (Antioquia, Colombia). Deionized water was obtained from a milli-Q integral 5 water purification system (Millipore, Billerica, MA, USA). All other chemicals, solvents and reagents were of analytical grade.

### Samples treatment

#### Avocado seeds treatment

Avocado seeds were washed and blanched for 5 min at 96 °C and then cut into 2 mm slices in a vegetable processor (JAVAR, Colombia). After that, samples were stored frozen (− 20 °C) until processing.

#### Drying and grinding

The drying process was performed according to the methodology described by Saavedra et al. (2017) with slight modifications. The convective drying process of avocado seeds was carried out in a tunnel dryer (Model DIES TS-120, DIES, Itagui, Colombia). The drying area in the tray was approximately 748 cm^2^. The drying process was conducted by triplicate (Drying 1, 2 and 3) using a constant air velocity (1.9 m/s) at 60 °C. The thickness of the seeds was 2 mm. Seeds were placed in a single tray, and drying time was brought up to constant sample weight. The weight difference was gravimetrically measured (Traveler Ohaus, TA502, New Jersey, USA) with a precision of 0.01 g. The variation of the avocado seeds moisture rate (MR) was determined using the equation described by Ramírez et al. (2019) (Eq. 1):

1 $${M}_\text{R}=\frac{{X}_\text{t}-{X}_{e}}{{X}_\text{o}-{X}_\text{e}},$$

where X_t_ is the moisture content at any time, X_e_ is the equilibrium moisture and X_o_ is the initial moisture content. All the results are expressed as g water/g dry matter. Due to X_e_ values are relatively small with respect to X_t_ or X_o_, the equation could be simplified as (X_t_/X_0_) (Duarte et al., 2017).

To determine the effective diffusion coefficients (Deff), the solution of Fick’s second law was used for an infinite plate (Eq. 2) (Osorio-Arias et al., 2019). The solution used was for moisture transport in only one direction, assuming that the process only occurs by diffusion. Furthermore, it is assumed that both diffusion coefficients and temperatures are constant.

2 $$ \text{MR}_\text{t}=\frac{8}{{\pi }^{2}}\sum_{i=1}^{\infty }\frac{1}{{(2i-1)}^{2}}{e}^{\left(\frac{-{(2i-1)}^{2}{\pi }^{2}{D}_{eff}t}{4{L}^{2}}\right)}$$

For long drying times, only the first term of Eq. 2 is necessary. This equation can be reduced as shown in Eq. 3:

3 $$ {\text{MR}}_{{\text{t}}}  = \frac{8}{{\pi ^{2} }}e^{{\left( {\frac{{ - {\text{Deff}} \times \pi ^{2}  \times t}}{{4L^{2} }}} \right)}}  $$

*D*_eff_ is the effective coefficient of diffusion for moisture (m^2^/s), t is the drying time expressed in seconds (s), and *L* is the average thickness of the sheet (m).

Once the avocado seed sheets were dry, they were submitted to a milling process in a POLIMIX PX-MFC 90D hammer mill (Kinematica/Lucerna, Switzerland) with a 1 mm sieve. Finally, they were vacuum-packed and stored at room temperature in darkness until their subsequent treatment.

#### Starch extraction

Starch extraction from the avocado seeds was performed according to Chel-Guerrero et al. (2016) with slight modifications. Initially, the ground powder was passed through an 80 mesh screen, using a Ro-Tap shaker for 10 min. The sieved powder was brought into a solution with sodium bisulfite (1500 ppm) with a 1/5 (m/v) ratio, which was stirred in a Shaker (Heidolph Unimax 1010, Schwabach, Germany) at 200 rpm for 1 h. After that, the suspension was passed through a cloth screen to separate the fibrous part, facilitate the screen’s filtration process, and avoid its plugging. Then it was again passed through the 80 mesh screen. The filtrate was allowed to settle for 5 h, and the supernatant was removed. The solid fraction was washed four times with distilled water and centrifuged in a centrifuge Boeco C-28A (Hamburg, Germany) at 4500 rpm for 8 min. The starch residue was dried at 40 °C for 12 h in a natural convection air oven (Binder Classic Line Model ED 23, Tuttlingen, Germany). Once dry, the starch was ground, sieved on a 20 mesh, and stored in a fully sealed glass container until analysis. To determine the percentage of starch extraction, the Eq. 4 was used:

4 $$ \% {\text{Starch}}\,{\text{extraction}}\, = \,\frac{{{\text{Dried}}\,{\text{Mass}}\,20\,{\text{mesh}}}}{{{\text{Initial}}\,{\text{Mass}}}}, $$

where Dried Mass 20 mesh is the dried mass obtained from starch once finalized the process. Initial mass $:$ is the mass of avocado seed to start the drying process.

#### Starch purity determination

The purity of the starch was carried out following the phenol–sulfuric acid method (Dubois et al., 1956). Briefly, 1 mL of starch solution (1 mg/mL) was mixed with 0.1 mL of phenol (80 g/100 g) and left to rest for 15 min. Then 3 mL of concentrated sulfuric acid (97 g/100 g) were added and left again to rest for 15 min. Finally, the absorbance was read at 540 nm. The amount of total carbohydrates corresponding to the starch content (Nielsen, 2003) present in the samples was calculated from the standard glucose curve (0–1 mg/mL). The analysis was carried out in triplicate, and the results were expressed in g of starch/100 g of sample.

#### Starch characterization

##### Differential scanning calorimetry (DSC)

The differential scanning calorimetry was determined according to Chel-Guerrero et al. (2016). The analysis was carried out in a Differential Scanning Calorimeter (DSC) equipment (Q100/TA Instruments, New Castle, Delaware-USA), under a UHP nitrogen atmosphere. 12 mg of starch were mixed with 28 μL of distilled water and the hermetic aluminum capsule was sealed and left to rest for 2 h. Sample was heated from 5 to 180 °C at a heating rate of 10 °C/min, and finally, it was cooled down to 5 °C at the same rate.

##### Fourier transform infrared spectrophotometry (FTIR)

FTIR spectra were performed following the methodology described by Warren et al. (2016) with modifications. Two mg of avocado seed starch were used, and a KBr tablet was made in an approximate ratio of 100:2 KBr-sample. A pressure of 5 Tons was applied. Analysis was conducted with a Fourier Transform Infrared equipment (Spectrumone, Perkín Elmer, Waltham, Massachusetts, USA) with a DTGS detector (deuterated triglycine sulfate detector elements). The analysis conditions were as follows: temperatures of 22 °C, 120 scans, a resolution of 4 cm^−1^ and a wavelength range of 4000–4500 cm^−1^.

##### Starch gelatinization

Gelatinization analyses of the starch extracted from the avocado seeds were carried out in a Brabender Visco-Amylo-Graph-U (Martinsried, Germany), provided by the company Poltec S.A.S (La Estrella, Medellín, Colombia). 15 g of the sample were mixed with 100 mL of distilled water and heated from 30 to 93 °C at a heating rate of 7.5 °C/min. The sample was kept at this temperature for 5 min and then cooled to 50 °C at a rate of 7.5 °C/min (Wiesenborn et al., 1994). Results were expressed in Brabender Units (BU).

##### Scanning electron microscopy (SEM)

A JEOL JSM 6490LV scanning electron microscope (Akishima-Tokio, Japan) was used to analyze the avocado seed starch images. Samples were fixed on a graphite tape, impregnated with a layer of gold (Au) using a DENTON VACUUM Desk IV equipment, and subsequently analyzed in a high vacuum scanning electron microscope to obtain high-resolution images (Liu et al., 2009).

##### Coating development

The coating was prepared following the methodology proposed by Oriani et al. (2014) with some modifications. Initially, 13 g of the starch isolated from the avocado seeds was dissolved in 100 mL of distilled water under constant stirring and heating. When the solution reached a temperature of 50 °C, 1 g of glycerol was added and brought to a temperature of 80 °C, where it was kept for 5 min. Finally, the prepared coating was left in an ice bath for 5 min.

### Potatoes treatment

Potatoes were washed with distilled water, peeled, and cut manually with a knife in the shape of rectangles (length: 5.5 cm, width: 1.5 cm, and thickness: 0.9 cm). Subsequently, they were immersed in water at room temperature (25 ± 2 °C) for 5 min to remove the starch from the surface and then dried with absorbent paper before being fried.

#### Application of the coating

The edible coating was applied to the potatoes according to the methodology proposed by Trujillo-Agudelo et al. (2019). Briefly, samples were immersed in the coating for 15 s. Subsequently, potatoes were dried in an oven (Binder Classic Line, Model ED 23, Tuttlingen Germany) with natural convection for 40 min at 50 °C. Potatoes were moved in the tray every 20 min to achieve uniform drying in all samples. Each treatment was done by quadruplicate.

#### Frying process

The frying process was carried out following Trujillo-Agudelo et al. (2019) methodology with some modifications. An electric fryer (Hamilton Beach, Model 35,021, United States) with a capacity of 2 L was used. The ratio potato/oil was 230 g:1.5 L in all frying treatments. Both coated and uncoated (control) samples were fried for 7 min at 190 °C. French fries were dried with absorbent paper to remove excess oil, cooled at room temperature, packed in bags, vacuum-sealed, and stored at 8 ± 2 °C until analysis.

#### Physico–chemical characterization of coated (CFF), uncoated (UFF) and commercial (CoFF) French fries

For the characterization of the coated (CFF) and uncoated (UFF) French fries, as well as commercial French fries (CoFF), moisture and fat content were determined according to the AOAC (2005). Water activity (Aw) was measured in a hygrometer (AQUALAB-PRE, Pullman-USA) at 25 °C. Color measurements were made at room temperature using X-RITE equipment, model SP62S (Grand Rapids, Michigan, USA), with a D65 illumination. The color was measured on the surface of each potato with four repetitions.

The firmness analysis included control (uncoated) and coated fried potatoes, as well as commercial potatoes. It was determined using a texture analyzer (TAXT plus, Stable Microsystems), as Trujillo-Agudelo et al. (2019) described. All analyses were performed in quadruplicate.

#### Acrylamide determination

Acrylamide was determined as described by Mesías and Morales (2015). The grounded sample was weighed and mixed with water in polypropylene centrifugal tubes. Hexane was added to the tubes to remove the fat content of the French fries. Mixture was spiked with 100 μL of a 5 μg/mL [^13^C_3_]-acrylamide methanolic solution as internal standard and later homogenized (Ultra Turrax, IKA, Mod-T10 basic, Germany) for 10 min. Afterward, the sample was treated with Carrez I (15 g potassium ferrocyanide/100 mL water) and Carrez II (30 g zinc acetate/100 mL water) solutions and centrifuged (9000×*g* for 10 min) at 4 °C. Hexane was removed, and samples were cleaned up by using Oasis-HLB cartridges. The solution was filtered through a 0.45 μm filter into an amber lite LC–MS vial. Sample extracts and calibration standards were analyzed by LC–ESI–MS-MS, as described by Mesías and Morales (2015). Accuracy of the results was demonstrated for potato crisps and pre-cooked French fries in four proficiency tests launched by the Food Analysis Performance Assessment Scheme (FAPAS) program, yielding a z-score of − 0.2 (Test 3071, Feb–March 2017), − 0.3 (Test 3080, Feb–March 2018), 0.0 (Test 3085, Sep–Oct 2018), and 0.3 (Test 3089, Feb-2019). Precision (reproducibility) was lower than 10%, and recovery was between 84 and 109%. The limit of the quantitation was set at 20 μg/kg. Analyses were performed in triplicate, and results were expressed as μg/kg of product.

### Sensorial analysis

The sensory evaluation was carried out in the Sensorial Food Analysis Laboratory of the University of Antioquia. The analysis was conducted with six trained panelists: five women and one man, aged between 25 and 60 years, The test used was a sensory profile by multidimensional approach according to NTC 3932 of 1996 (Instituto Colombiano de Normas Técnicas y Certificación, 1996) and NTC 3501 of 2012 (Instituto Colombiano de Normas Técnicas y Certificación, 2012), where descriptors of appearance, smell, taste, and texture were evaluated taking into account a rating scale of 0–5 (0 was absent and 5 was very intense). An acceptance scale of 1–3 was established for the general quality descriptor, where 1 was the lowest quality and 3 was the highest.

### Statistical analysis

A randomized experimental design was applied to evaluate the application of the edible coating on the reduction of acrylamide and fat levels in French fries. The Independent variable was the coating (coated, uncoated, and commercial samples), and moisture, firmness, fat, color, and acrylamide content were analyzed as response variables. Analysis of variance (one-way ANOVA) with a level of significance of 95% and with Fisher LSD’s multiple comparisons post hoc test was used. The homogeneity of variances was determined with Levene’s test and the Kolmogorov–Smirnov test was applied to check the Lack of fit. All statistical analyses and graphic illustrations were studied using the statistical software Statgraphics Centurion XVI (Statgraphics Technologies, Inc, Virginia, USA).

## Results and discussion

### Drying process

**Fig. 1:**
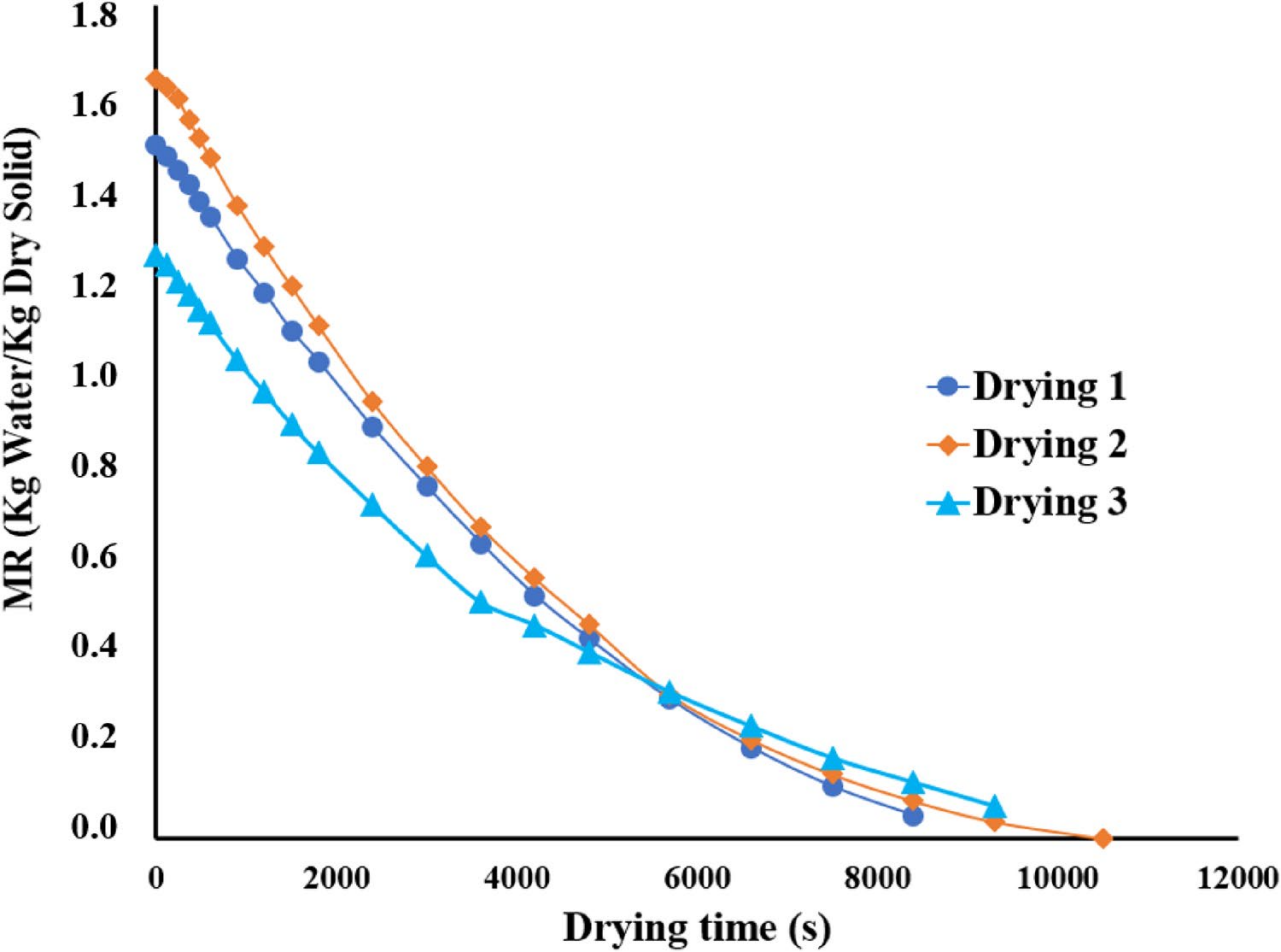
Drying curve for avocado seeds

Figure 1 depicts the kinetics of drying for the slices of avocado seeds. The samples initial moisture content of the samples varied from 56 to 62%, which is in line with the range from 55 to 65% reported by Saavedra et al. (2017). The drying time ranged from 8400 and 10,500 s, reaching final moisture rates of 0.037 to 0.070 kg water/kg dry solid, which means a moisture content ranging from 3.55 to 6.52%. The literature describes similar results in avocado seeds for convective and microwave drying methods (Araujo et al., 2020; Saavedra et al., 2017). Diffusion coefficients based on the Fick model were between 4.86 and 6.48 × 10^–10^ m/s. The values are similar to those determined by Saavedra et al. (2017) but lower than those reported by Araujo et al. (2020). The data agreed with those for other agro-industrial waste treated with drying processes, such as passion fruit peel (Duarte et al., 2017) or spent coffee grounds (Osorio-Arias et al., 2019). Overall, the order of magnitude of diffusion coefficients was between 10^–9^ and 10^−12^ m^2^/s, which are dependent on the food matrix and the drying process conditions.

### Starch characterization

Before describing the properties of starch extracted from avocado seed, it is worth mentioning that 13 kg of avocado seed gave rise to 1.5 kg of starch, which meant a process yielding 11.53% according to Eq. 4. The proportion of starch in an avocado seed is usually around 29% (Chel-Guerrero et al., 2016). However, our result is closer to that established by Macena et al. (2020), who achieved a starch extraction of 10%.

The starch purity was 41.8 g/100 g of sample. This value is lower than that reported for other types of starches. In general, starch purity depends on the food matrix and the quantification method. In this sense, starch purities of 89% has been reported in rice (Rodriguez, 2015), 90% in Chinese potatoes (*Colocasia esculenta*) (Hurtado, 2019), 91% in corn (*Zea mays*) (Yugsi, 2017), 95% in potatoes (Yugsi, 2017), and 99% in jackfruit seeds (*Artocarpus heterophyllus* Lam.) (Zhang et al, 2016). It is noteworthy that to our knowledge, the purity of starches extracted from avocado seed had not been reported yet. An insoluble residue was observed during the purity analysis, indicating the presence of fiber from the seed. This finding would explain the lower purity level reached as compared with literature for other types of starch.

#### Differential scanning calorimetry (DSC)

**Fig. 2:**
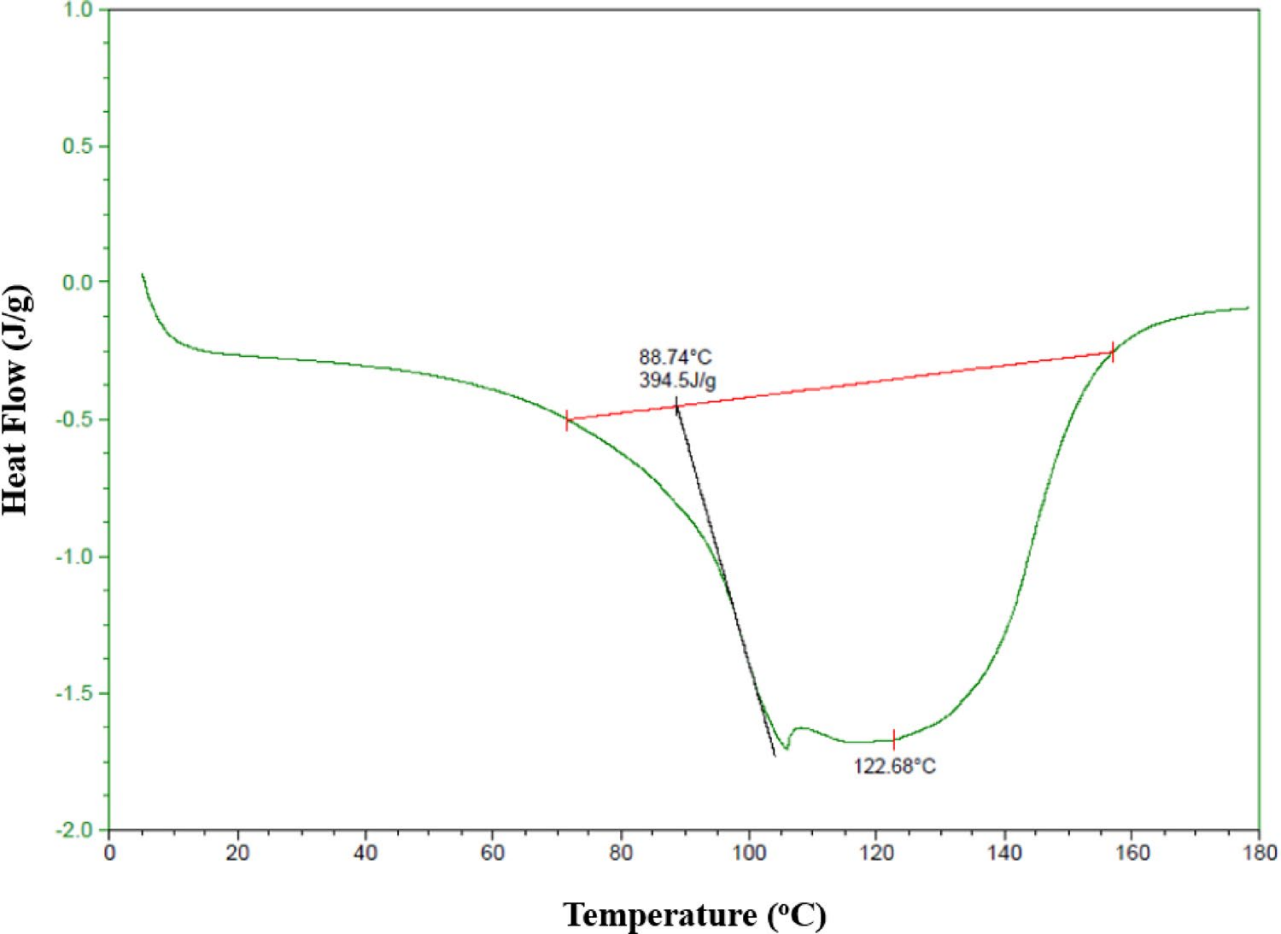
Thermogram obtained for the starch extracted from avocado seeds

Figure 2 shows the thermogram recorded for the starch extracted from the avocado seeds, where a fusion temperature of 122.68 °C and an enthalpy of 394.5 J/g were observed. The fusion temperature suggests that double-helix chains of amylopectin in the extracted starch have a great crystalline structure, which depicts higher stability to the thermal processes (Li et al., 2020a). The high degree of crystallinity in the starch is related to the enthalpy value, pointing that high energy is required to reach a phase change (Builders et al., 2010). Builders et al. (2010) showed fusion temperatures of 295 °C in the starch also coming from avocado seeds, where a crystalline structure similar to the starch corn was found. The different fusion temperatures observed in our study could be attributed to changes in the chemical composition of starches linked to their origins and varieties (Pardo et al., 2013). Fusion temperatures similar to the present research have been stated for different types of resistant starches (Liu et al., 2020), corn and sweet potato starch mixtures (Ding et al., 2020), and corn starch (Doblado-Maldonado et al., 2016).

#### Fourier-Transform Infrared Spectroscopy (FTIR)

FTIR analysis of the starch from avocado seeds. The typical bands corresponding to the functional groups of starch are observed at wavelength 2923 cm^−1^, 1156.77 cm^−1^, and 861.23–1079.54 cm^−1^, for C–H, C–O, C–C, and C–C C–O–H, respectively (Warren et al., 2016). FTIR bands at 1000 and 1047 cm^−1^ correspond to crystalline structures are shown, whereas amorphous chains locate at 1023 cm^−1^ (Bello-Pérez et al., 2005). According to Warren et al. (2013), the FTIR band at 1000–1022 cm^−1^ is more related to the crystallinity of starch, which can also be evidenced in the DSC analysis.

#### Starch gelatinization

**Fig. 3:**
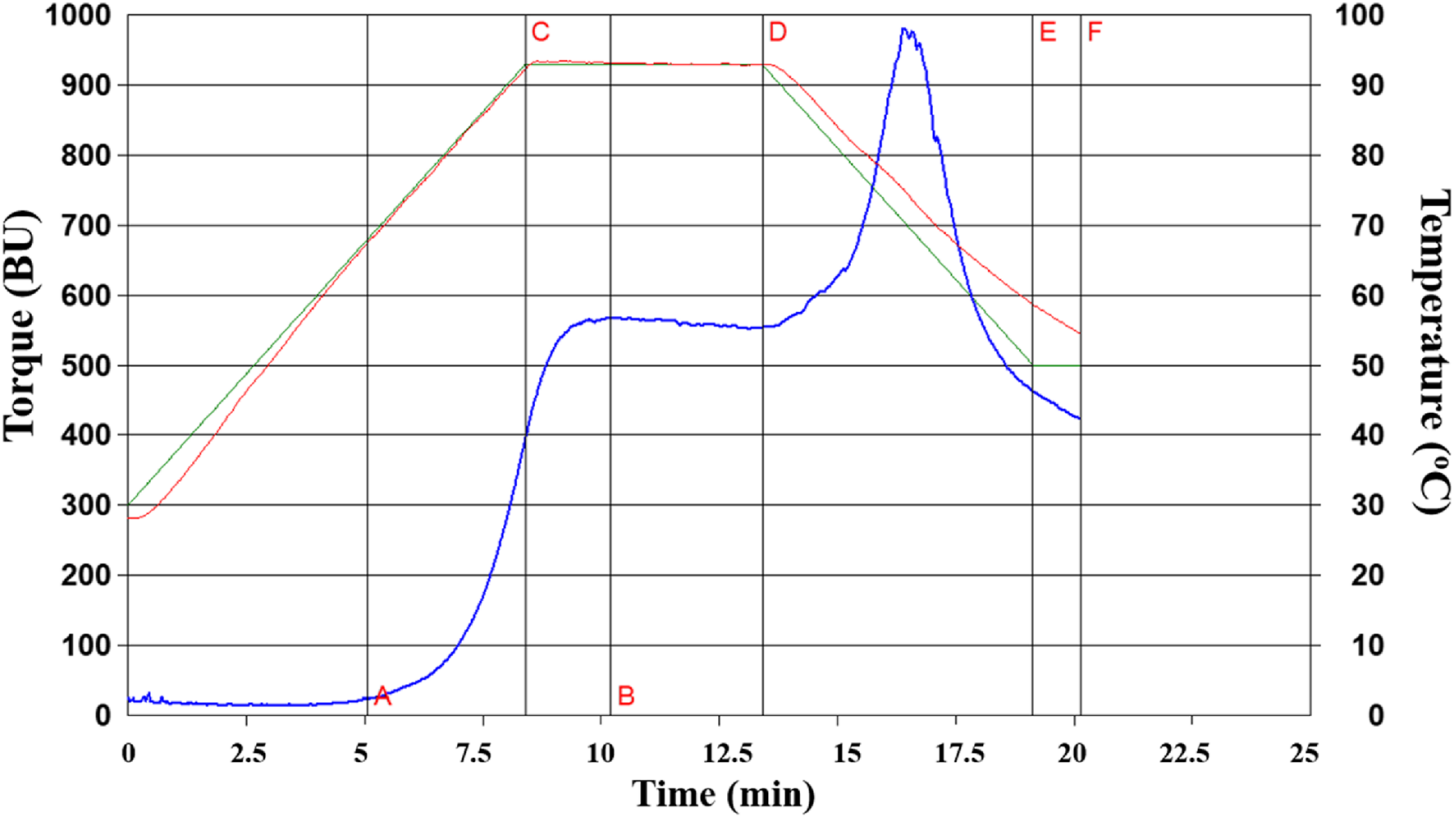
Visco-amylogram of the starch extracted from avocado seeds

The pasting and mechanical properties of the starch from avocado seeds during thermal treatment, such as gelatinization, swelling, viscosity increase, among other, were recorded from the resulting amylograms (Fig. 3). The maximum viscosity was 568 *BU* at 93.2 °C. Once the cooling period was started, viscosity raised to 950 *BU*. This fact could be attributed to starch retrogradation, a process in which amylose and amylopectin get reorganized due to the decline in temperature and formation of bonds between hydrogen molecules (Lacerda et al., 2014). Starch consistency was reached about 250 *BU*. Based on these data, we can conclude that the avocado seed starch obtained in the present study had good stability against mechanical works and thermal treatments (Osundahunsi et al., 2003). Values of 95 °C have been documented as temperatures for maximum viscosity in starch samples coming from avocado seeds (Lacerda et al., 2014; Chel-Guerrero et al., 2016), close to results observed in this study. Similarly, other types of starch extracted from sources such as yucca (Lacerda et al., 2014) and sorghum (Rivera-Corona et al., 2014) have shown similar thermal behaviors, with slight variation in the temperature for the maximum starch viscosity.

#### Scanning electron microscope (SEM)

**Fig. 4:**
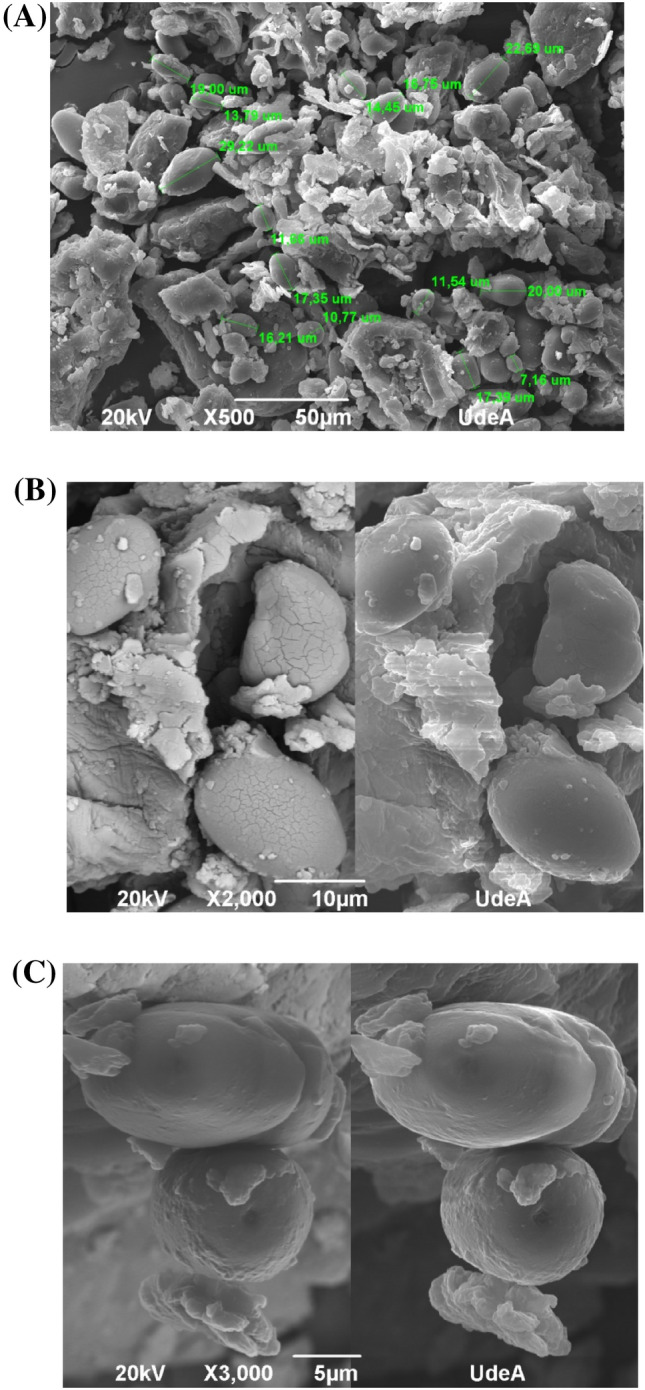
Scanning Electron Microscope (SEM) micrographs of the starch extracted from avocado seeds: Starch granule sizes (**A**), corrugated surfaces (**B**) and irregular, smooth and depressed surfaces (**C**)

SEM micrographs for the starch from avocado seeds are shown in Fig. 4. Micrographs depict oval and spherical shapes, with smooth and corrugated surfaces and a medium-size between 10 and 22 μm (Fig. 4A). These data are in accordance with results reported by Builders et al. (2010), who classified the particle size in the same matrix as a medium magnitude.

The presence of smooth structures would point to a good purity in the starch (Oyeyinka et al., 2020), whereas the corrugate surfaces could be due to damages induced during the extraction procedure or grinding (Fig. 4B). Lastly, Fig. 4C depicts some starch granules that have an uneven, smooth, and depressed surface. This could indicate that, although extracted starch was pure enough, the extraction and washing procedures should be improved. Similar shapes, morphologies, and sizes have been described for different types of starches, such as potatoes (Kumar et al., 2020) or yucca (Oyeyinka et al., 2020).

### Physico–chemical characterization of coated, uncoated and commercial French fries

Commercial (CoFF) and experimental French fries, coated (CFF) or uncoated (UFF) with starch from avocado seeds, were physicochemically characterized by determining moisture, water activity, fat content, color, and firmness (Table 1).

**Table 1:** Physicochemical characterization of uncoated (UFF), coated (CFF) and commercial (CoFF), French fries

Samples	Moisture (%)	Aw	Fat (g/100 g)	L*	a*	b*	Firmess (N)	Acrylamide (µg/kg)
UFF	48.89 ± 0.62^a^	0.884 ± 0.002^a^	7.84 ± 1.52^a^	48.94 ± 5.20^b^	-0.03 ± 1.147^a^	14.08 ± 1.51^a^	1.18 ± 0.16^a^	186 ± 4^b^
CFF	49.44 ± 1.42^a^	0.889 ± 0.001^b^	6.81 ± 0.53^a^	37.37 ± 4.33^a^	5.44 ± 1.39^b^	17.03 ± 0.48^b^	1.10 ± 0.14^a^	99 ± 2^a^
CoFF	49.36 ± 0.73^a^	0.884 ± 0.002^a^	10.49 ± 0.68^b^	63.02 ± 1.34^c^	-1.06 ± 0.10^a^	20.89 ± 1.27^c^	0.76 ± 0.31^b^	163 ± 7^c^
*p* value Kolmogorov–Smirnov test	0.941	0.838	0.630	0.851	0.353	0.990	0.917	0.753

Different letters mean significant differences (*p* < 0.05) between averages for UFF, CFF and CoFF

Mean values for moisture content in CoFF, UFF and CFF were 49.36, 48.89, and 49.44%, respectively, without significant differences among them (*p* > 0.05). The moisture was not affected by the type of treatment applied, probably due to frying temperature did not modify the water evaporation rate. In addition, experimental samples exhibited similar moisture content to commercial potatoes. Values ranging from 49 to 60% for moisture in French fries have been reported by other authors (Bingol et al., 2012; Li et al., 2020b). Conversely, Aw presented significant differences (*p* < 0.05), between treatments CFF–CoFF and CFF–UFF. The highest magnitude was reached in CFF (0.889), which was also the sample with greater moisture content. This could be attributed to the gelling that occurred in the starch of the coating during the beginning of the frying process, a fact preventing the water leakage (Mousa, 2018). No significant differences were found between CoFF and UFF treatments (*p* > 0.05).

The fat content in experimental samples (*p* < 0.05) was significantly lower than in commercial potatoes. Although without significant differences (*p* > 0.05), between CFF and UFF treatments, the fat content in CFF and UFF was 6.81 g/100 g and 7.84 g/100 g, respectively. The lower fat content in coated French fries as compared with CoFF samples could be due to two reasons: (1) the starch in the film acts as a lipid barrier; (2) the starch gelling during the drying process before frying, as a consequence of heating (Kurek et al., 2017). Another possible reason for the smaller fat level could be the high moisture content of potatoes since fat absorption only takes place at low moisture rates (Angor et al., 2013). Some investigations have reported that coatings decrease the fat content of fried products (Trujillo-Agudelo et al., 2019) and establish that the moisture content is inversely correlated with the fat level of French fries or chicken nuggets (Li et al., 2020b).

Color parameters in French fries evidenced statistically significant differences (*p* < 0.05) among the experimental potatoes and the commercial samples. It is known that luminosity decreases during thermal processing due to the changes in the morphology of the food surface. This phenomenon is related to the water leakage at the beginning of the frying, which leads to a higher light scattering and, therefore, making L* values decreased (Bingol et al., 2012). L* is also reduced as time and temperature of frying increase due to the non-enzymatic browning (Pedreschi et al., 2007). In parallel, positive b* values and low a* values indicate that fried potatoes acquire a yellow–brown coloration, characteristics preferred by most consumers (Bingol et al., 2012). In the present study, coated French fries had the lowest L* value (37.37) and the highest a* value. These results may be due to the red-brown color of the extracted starch or even to a possible conversion of chlorophyll to pheophytin during the frying process (Bingol et al., 2012) more than to a higher non-enzymatic browning, since despite the highest dark color, CFF exhibited the lowest concentration of acrylamide, as mentioned later. Chel-Guerrero et al. (2016) found that the native avocado starch was an opaque light-brown paste when fully gelatinized, whereas other native starches form colorless translucent pastes. Another hypothesis would be that coating promoted a higher browning only at the surface of the French fries, protecting the product from the development of Maillard reaction inside the potato during frying. Similar color parameters have been described in other investigations carried out in French fries (Bingol et al., 2012; Li et al., 2020b).

#### Firmness analysis

Firmness analysis in French fries showed mean values of 0.76, 1.18, and 1.10 for CoFF, UFF, and CFF, respectively. However, UFF and CFF did not present significant differences; experimental treatment provided significantly more firmness to the French fries than commercial samples (*p* < 0.05). However, and according to Bourne (2002), the values detected would point out that fried potatoes presented a low stiffness state and high weakness, the last related to their higher moisture content. Additionally, the low firmness values could be due to a possible gelling of the starch contained in the potatoes at the beginning of the frying process (Bingol et al., 2012). The firmness values obtained are similar to those reported by Trujillo-Agudelo et al. (2019), Bingol et al. (2012), and Li et al. (2020b) in French fries. In contrast, higher data in the range of 3–5 N have been detected by Morales et al. (2014).

#### Acrylamide content

Acrylamide content in French fries is compiled in Table 1. All the samples were below the benchmark level established in the UE 2158/2017 Regulation for French fries (750 μg/kg) (European Commission, 2017). The acrylamide content in the French fries was significantly affected by the coating (*p* < 0.05). Acrylamide concentration was significantly decreased in CFF, exhibiting levels 45% lower than uncoated samples (99 *vs.* 186 µg/kg, respectively) and lower than CoFF (99 *vs.* 163 µg/kg, respectively). Different reasons could justify the decrease in the acrylamide formation in potato fried products coated with starches. Firstly, starch could form protecting films on the surface of the products due to gelling processes taking place above 60 °C. These films could prevent water leakage, avoiding oil penetration in the food surface. In addition, the starch coating could block molecular reactions leading to acrylamide formation (Mousa, 2018; Kurek et al., 2017). This fact can be supported by the higher Aw (0.889) detected in CFF, which did not promote the development of the Maillard reaction (Gómez-Narváez et al., 2019). Several authors have reported reductions ranging from 38 to 73% in the acrylamide content in French fries with edible coatings (Jung et al., 2003; Urbancic et al., 2014; Trujillo-Agudelo et al., 2019).

#### Sensory analysis

Scores recorded for sensorial descriptors of appearance (color uniformity), smell, taste, and texture for uncoated (UFF) and coated (CFF) French fries, as well as the global quality for each one of them are depicted in Table 2. A statistically significant difference was detected between both types of fried potatoes in the color uniformity, whereas UFF exhibited the highest value (4.4 *vs*. 4.0 for UFF and CFF, respectively; *p* < 0.05). For smell descriptors such as oily, sweet, fresh, and saline, the greatest score was reached in the UFF, whereas the roasted smell was best valued in the CFF. In the case of sensory descriptors for taste oily, sweet, fresh, saline, and roasted, the UFF got the best ratings (*p* < 0.05). It is worthy of mentioning that the umami, vegetable and green taste, and the somatosensory sensations astringent and spicy were more intense in the CFF. The umami taste is due to the presence of precursors in the potato such as glutamic acid, aspartic acid, guanosine 5′-monophosphate (GMP), and adenosine 5′-monophosphate (AMP), which are enhanced during the cooking method (Raigond et al., 2015) producing the perception of the umami attribute. Concerning texture parameters, the chewiness, cohesivity, and crispness of CFF reached higher scores. The overall sensory quality of French fries was high and similar for both samples, with a mean value of 3.0 (*p* > 0.05). From these results, it could be admitted that potatoes are accepted with good sensory perception (CFF and UFF) since, according to the judges, they had the highest score from the sensory quality.

**Table 2:** Sensorial descriptors of appearance (color uniformity), smell, taste, and texture for uncoated (UFF) and coated (CFF) French fries

Descriptor	UPC	CPC	*p* value
Color uniformity	4.4 ± 0.2	4.0 ± 0.0	^++^
Smell			
Oily	4.0 ± 0.0	3.4 ± 0.2	^++^
Sweet	1.9 ± 0.2	1.0 ± 0.0	^++^
Fresh	4.5 ± 0.0	3.5 ± 0.0	^++^
Saline	1.8 ± 0.3	1.0 ± 0.0	^++^
Roasted (Frying)	3.3 ± 0.3	4.0 ± 0.0	^++^
Taste			
Oily	2.9 ± 0.2	2.5 ± 0.0	^++^
Bitter	0.5 ± 0.0	1.0 ± 0.0	^++^
Sweet	1.0 ± 0.0	0.6 ± 0.2	^++^
Fresh	4.5 ± 0.0	4.0 ± 0.0	^++^
Saline	0.8 ± 0.3	0.6 ± 0.2	^++^
Roasted (Frying)	1.5 ± 0.0	1.2 ± 0.4	^++^
Umami	0.0 ± 0.0	1.0 ± 0.0	^++^
Vegetable	0.5 ± 0.0	1.0 ± 0.0	^++^
Green	0.0 ± 0.0	1.0 ± 0.0	^++^
Astringent	0.3 ± 0.3	1.3 ± 0.3	^++^
Spicy	0.9 ± 0.2	1.5 ± 0.0	^++^
Texture			
Oily	2.9 ± 0.2	2.5 ± 0.0	^++^
Adhesive	1.1 ± 0.2	0.5 ± 0.0	^++^
Cohesive	1.5 ± 0.0	2.0 ± 0.0	^++^
Crispy	1.5 ± 0.0	2.0 ± 0.0	^++^
Hard	1.9 ± 0.2	1.1 ± 0.2	^++^
Lumpy	2.3 ± 0.3	1.9 ± 0.2	^++^
Chewy	2.0 ± 0.0	2.5 ± 0.0	^++^
Global quality	3.0 ± 0.0	3.0 ± 0.0	--

^++^Significant differences between uncoated and coated samples (*p* < 0.05)

Double hyphen indicates not significant differences between uncoated and coated samples (*p* > 0.05)

In the present study, it has been revealed that convective drying processes are a good alternative for agroindustrial waste from the avocado industry. The drying kinetic of avocado seeds evidenced an optimal behavior according to the Fick’s second law. Therefore, a dehydrated seed suitable for subsequent industrial processing was produced. Results demonstrated that avocado seed is an alternative and inexpensive source of starch, which depicts proper stability to the mechanical works and thermal treatments and, consequently, it could be satisfactorily used in the food industry. The starch coating may be an effective strategy to reduce fat content and mitigate acrylamide formation during potato frying, providing a protective effect that avoids water leakage and oil penetration in the potato during frying. Moreover, despite some modifications in the sensory profile of coated French fries, the final product shows optimal organoleptic attributes and consumer acceptability.
